# Supplementary material for: Convergence of aging- and rejuvenation-related epigenetic alterations on PRC2 targets
Source: Mol Syst Biol. 2026 Feb 10;22(5):787–810. doi: 10.1038/s44320-026-00195-9 (PMC13144388; doi:10.1038/s44320-026-00195-9)
Supplement: Supplementary file 1 — Appendix [file 44320_2026_195_MOESM1_ESM.pdf]

Appendix for

Convergence of aging- and rejuvenation-related epigenetic alterations on PRC2 targets

Oscar Camacho[1,†], Michael A. Koldobskiy[1,2,†,*], Pradeep Reddy[3,4], Atharv Oak [1], Yuxiang Sun[5], Kenna Sherman[1,6], Juan Carlos Izpisua Belmonte[3,4,*] and Andrew P. Feinberg[1,6,7,8,*]

[1] Center for Epigenetics, Johns Hopkins University School of Medicine, Baltimore, MD, USA

[2] Pediatric Oncology, Sidney Kimmel Comprehensive Cancer, Baltimore, MD, USA

[3] Salk Institute for Biological Studies, La Jolla, CA, USA

[4] Current Address: Altos Labs, San Diego, CA, USA

[5] Department of Nutrition, Texas A&M University, College Station, TX, USA

[6] Department of Genetic Medicine, Johns Hopkins University School of Medicine, Baltimore, MD, USA

[7] Departments of Medicine, Biomedical of Engineering and Public Health, Johns Hopkins University, Baltimore, MD, USA

[8] Department of Biomedical Engineering, Tel Aviv University, Tel Aviv, Israel

† These authors contributed equally.

* To whom correspondence should be addressed. Email: mak@jhmi.edu, jcbelmonte@altoslabs.com, and afeinberg@jhu.edu

<u>Table of Contents</u>

Name	Page
Appendix Figure S1	2-5

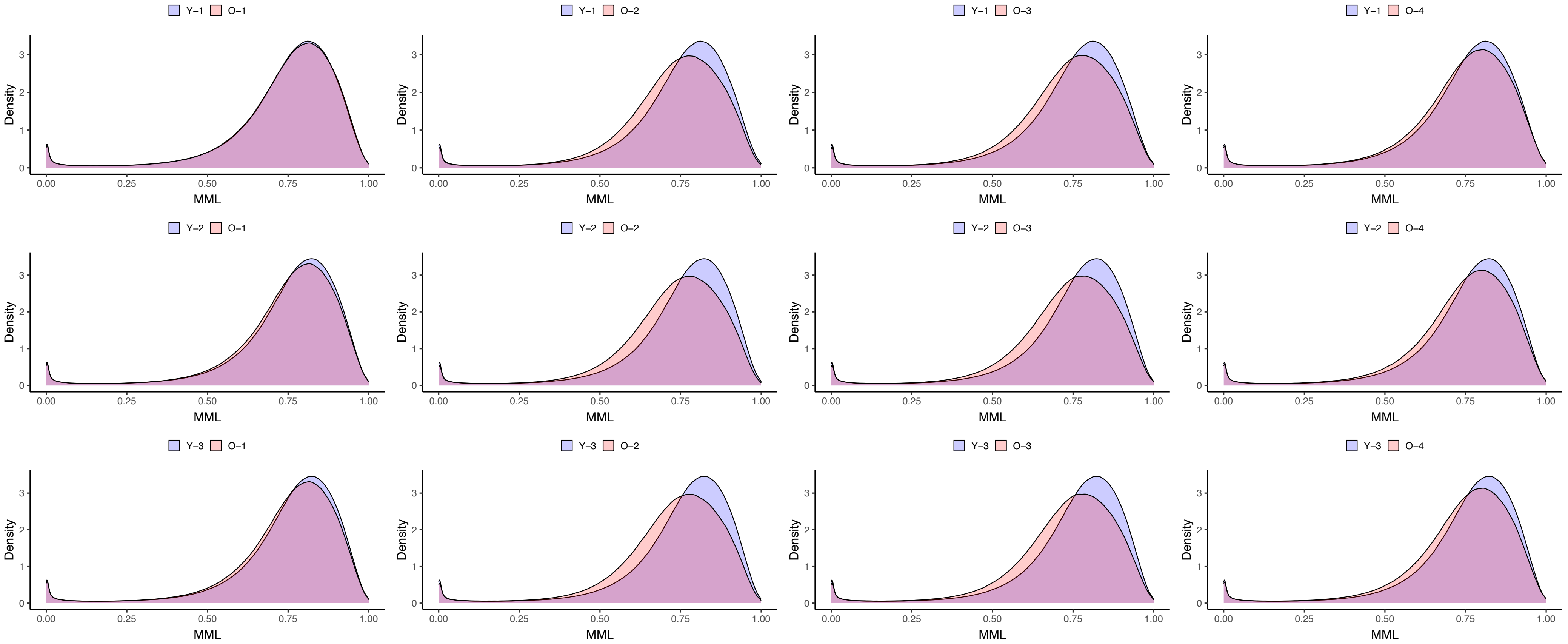
Y–1 O–1
Y–1 O–2
Y–1 O–3
Y–1 O–4
Y–2 O–1
Y–2 O–2
Y–2 O–3
Y–2 O–4
Y–3 O–1
Y–3 O–2
Y–3 O–3
Y–3 O–4
Density
0
1
2
3
MML
0.00
0.25
0.50
0.75
1.00

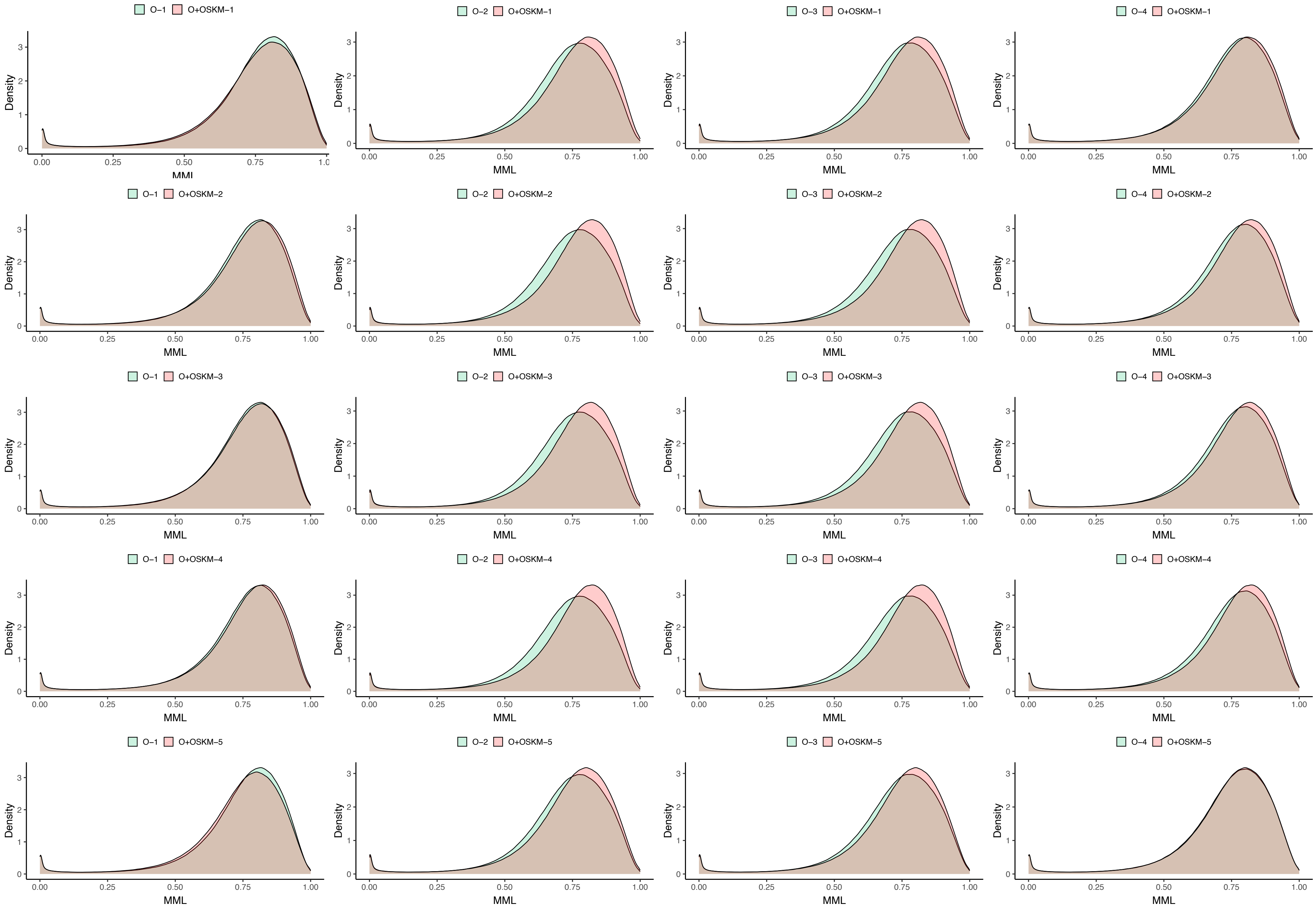
O−1 O+OSKM−1
O−2 O+OSKM−1
O−3 O+OSKM−1
O−4 O+OSKM−1
O−1 O+OSKM−2
O−2 O+OSKM−2
O−3 O+OSKM−2
O−4 O+OSKM−2
O−1 O+OSKM−3
O−2 O+OSKM−3
O−3 O+OSKM−3
O−4 O+OSKM−3
O−1 O+OSKM−4
O−2 O+OSKM−4
O−3 O+OSKM−4
O−4 O+OSKM−4
O−1 O+OSKM−5
O−2 O+OSKM−5
O−3 O+OSKM−5
O−4 O+OSKM−5
Density
MML
0.00 0.25 0.50 0.75 1.00
0 1 2 3

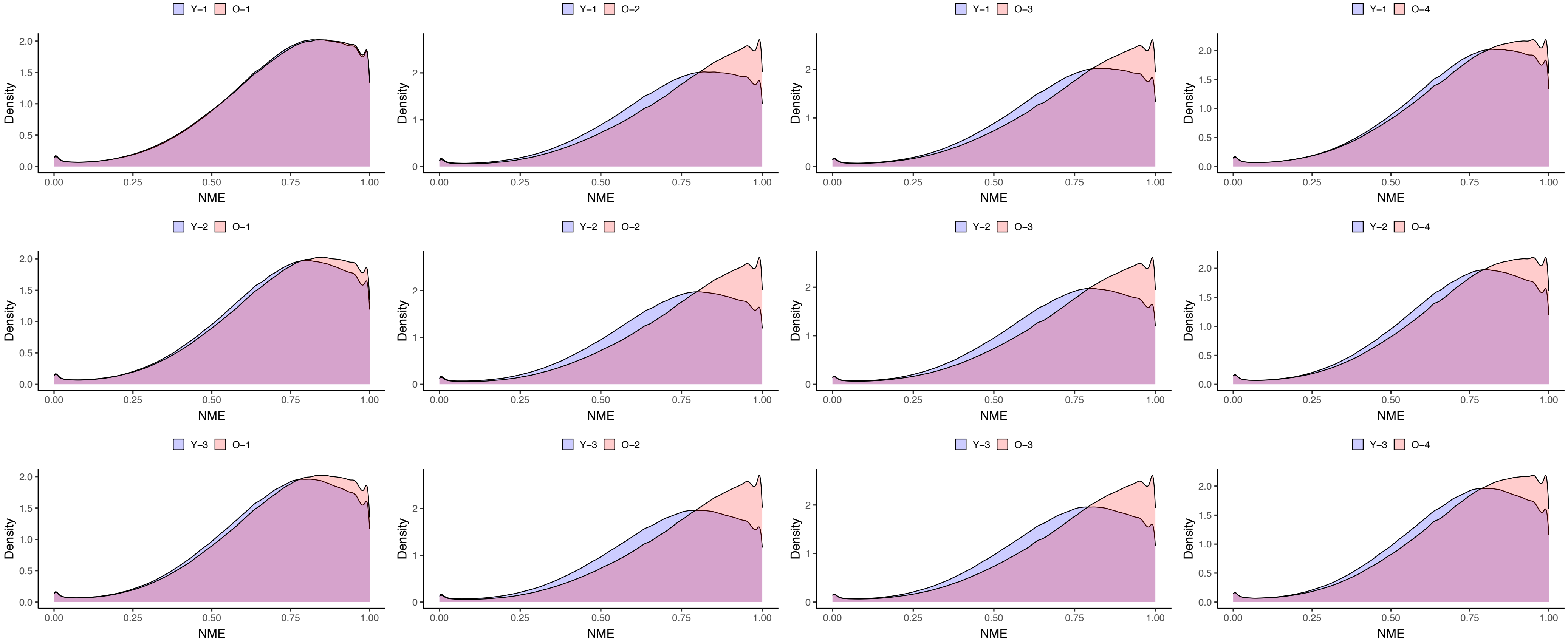
Y–1 O–1
Y–1 O–2
Y–1 O–3
Y–1 O–4
Y–2 O–1
Y–2 O–2
Y–2 O–3
Y–2 O–4
Y–3 O–1
Y–3 O–2
Y–3 O–3
Y–3 O–4
Density
NME
0.00 0.25 0.50 0.75 1.00
0.0 0.5 1.0 1.5 2.0
0 1 2

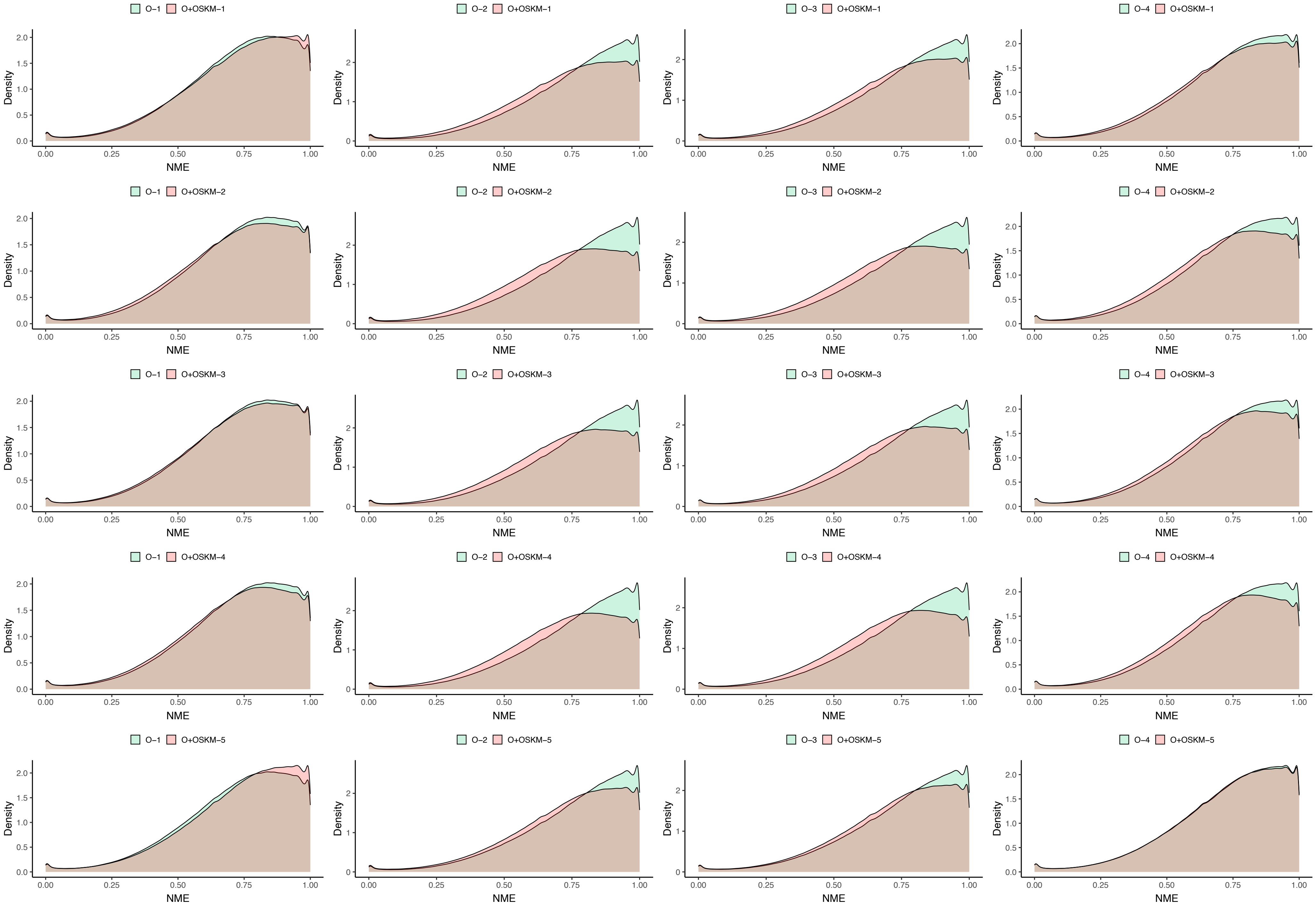

Appendix Figure S1. Density plots of genome-wide distributions of mean methylation level (MML) and normalized methylation entropy (NME) from pairs of young (Y), old untreated (O), and old treated (O+OSKM) dorsal skin WGBS samples.
