# Supplementary material for: Convergence of aging- and rejuvenation-related epigenetic alterations on PRC2 targets
Source: Mol Syst Biol. 2026 Feb 10;22(5):787–810. doi: 10.1038/s44320-026-00195-9 (PMC13144388; doi:10.1038/s44320-026-00195-9)
Supplement: Supplementary file 13 — Expanded View Figures [file 44320_2026_195_MOESM13_ESM.pdf]

Expanded View Figures

Figure EV1. Global DNA methylation patterns and sample composition in whole skin WGBS.

(**A**) PCA plot of genome-wide MML in WGBS samples from whole skin, including Young, Old untreated (Old) and Old treated (Old+OSKM) samples. (**B**) Box plots of MML and NME distributions within selected genomic regions. For each box plot, the central line represents the median, the box bounds correspond to the first (Q1) and third quartiles (Q3), and the whiskers extend to the most extreme data points within 1.5× the interquartile range (IQR) from the quartiles. Student's *t* tests were performed using the average MML or NME value of each independent sample and comparing groups Young ($n = 3$), Old ($n = 4$) and Old+OSKM ($n = 5$). MML comparisons: Island: Old vs Young: $P = 0.325$; Old+OSKM vs Old: $P = 0.074$. Shore: Old vs Young: $P = 0.129$; Old+OSKM vs Old: $P = 0.395$. Shelf: Old vs Young: $P = 0.037$; Old+OSKM vs Old: $P = 0.076$. Open Sea: Old vs Young: $P = 0.040$; Old+OSKM vs Old: $P = 0.047$. Promoter: Old vs Young: $P = 0.046$; Old+OSKM vs Old: $P = 0.146$. Gene Body: Old vs Young: $P = 0.037$; Old+OSKM vs Old: $P = 0.034$. Exon: Old vs Young: $P = 0.032$; Old+OSKM vs Old: $P = 0.036$. Intron: Old vs Young: $P = 0.045$; Old+OSKM vs Old: $P = 0.071$. Intergenic: Old vs Young: $P = 0.039$; Old+OSKM vs Old: $P = 0.044$. NME comparisons: Island: Old vs Young: $P = 0.023$; Old+OSKM vs Old: $P = 0.025$. Shore: Old vs Young: $P = 0.042$; Old+OSKM vs Old: $P = 0.035$. Shelf: Old vs Young: $P = 0.038$; Old+OSKM vs Old: $P = 0.031$. Open Sea: Old vs Young: $P = 0.041$; Old+OSKM vs Old: $P = 0.029$. Promoter: Old vs Young: $P = 0.029$; Old+OSKM vs Old: $P = 0.028$. Gene Body: Old vs Young: $P = 0.032$; Old+OSKM vs Old: $P = 0.019$. Exon: Old vs Young: $P = 0.028$; Old+OSKM vs Old: $P = 0.022$. Intron: Old vs Young: $P = 0.048$; Old+OSKM vs Old: $P = 0.035$. Intergenic: Old vs Young: $P = 0.038$; Old+OSKM vs Old: $P = 0.029$. (**C**). Heatmap showing the proportion of latent DNA methylation components (LMC) across whole skin samples. LMCs and their proportions in samples were generated from decomposition of DNA methylation data by a reference-free cell-type deconvolution approach implemented by the MeDeCom software to estimate cell-type components (Lutsik et al, 2017). Most samples are primarily represented by a single LMC, indicating relatively homogeneous DNA methylation patterns and arguing against substantial cell-type admixture. Aging or rejuvenation treatment is associated with a transition toward a different dominant LMC, reflecting biological shifts in DNA methylation patterns rather than changes in cell-type composition.

 © The Author(s)

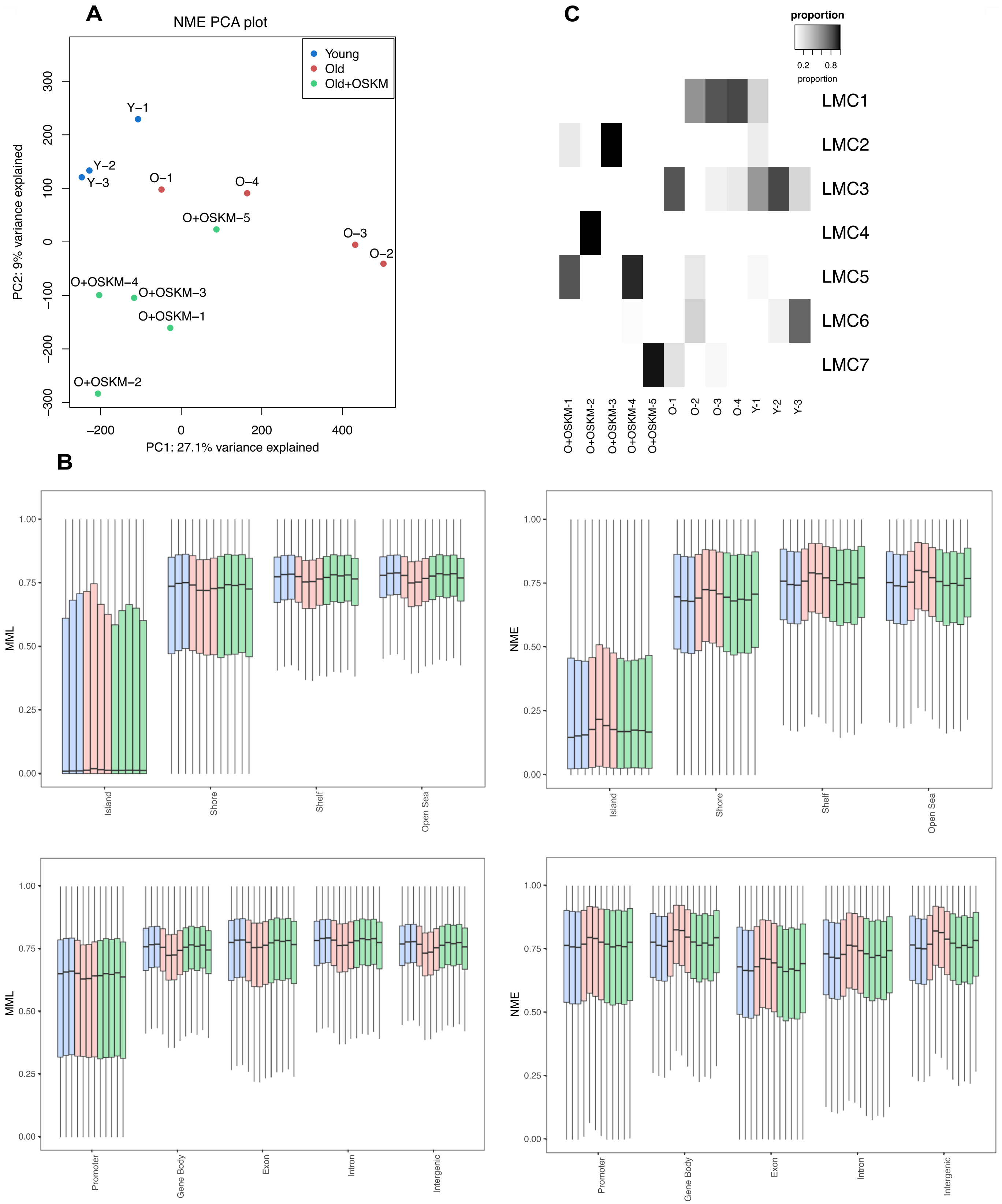
A
NME PCA plot
Young
Old
Old+OSKM
Y−1
Y−2
Y−3
O−1
O−2
O−3
O−4
O+OSKM−1
O+OSKM−2
O+OSKM−3
O+OSKM−4
O+OSKM−5
PC1: 27.1% variance explained
PC2: 9% variance explained
C
proportion
0.2 0.8
LMC1
LMC2
LMC3
LMC4
LMC5
LMC6
LMC7
O+OSKM-1 O+OSKM-2 O+OSKM-3 O+OSKM-4 O+OSKM-5 O-1 O-2 O-3 O-4 Y-1 Y-2 Y-3
B
MML
NME
Island
Shore
Shelf
Open Sea
Promoter
Gene Body
Exon
Intron
Intergenic

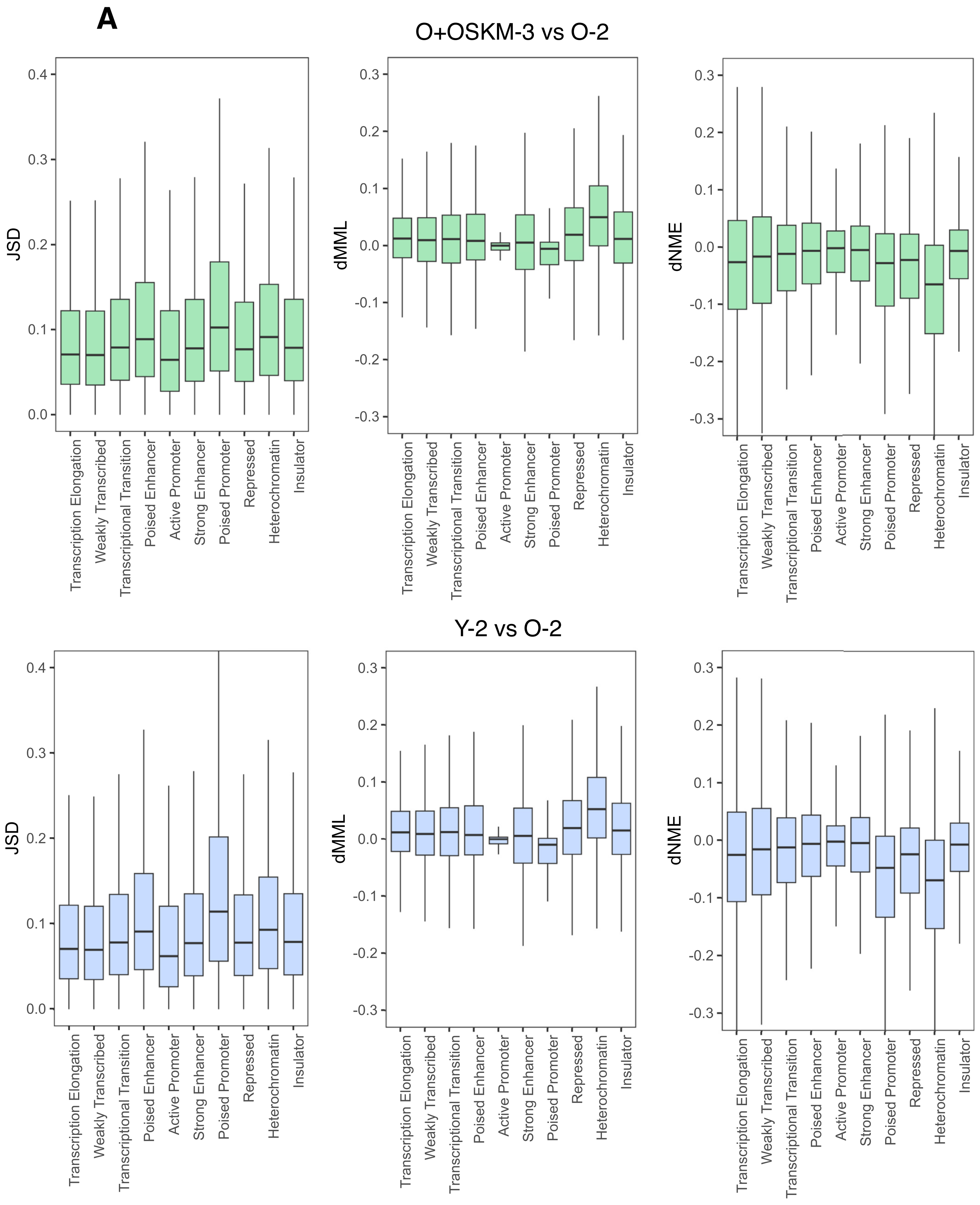
A
O+OSKM-3 vs O-2
Y-2 vs O-2
JSD
dMML
dNME
Transcription Elongation
Weakly Transcribed
Transcriptional Transition
Poised Enhancer
Active Promoter
Strong Enhancer
Poised Promoter
Repressed
Heterochromatin
Insulator

 © The Author(s)

Figure EV2. DNA methylation changes across chromHMM chromatin states in whole skin during aging and rejuvenation.

(**A**) Box plots of dMML, dNME, and JSD distributions across chromatin states defined by chromHMM (derived from mouse embryonic stem cells), based on per-region differences in representative pairwise comparisons between individual whole skin samples, including Young (Y-2), Old untreated (O-2), and Old treated (O + OSKM-3). Each box summarizes the distribution of values across genomic regions assigned to each chromatin state. For each box plot, the central line represents the median, the box bounds correspond to the first (Q1) and third quartiles (Q3), and the whiskers extend to the most extreme data points within 1.5× the interquartile range (IQR) from the quartiles.

© The Author(s)

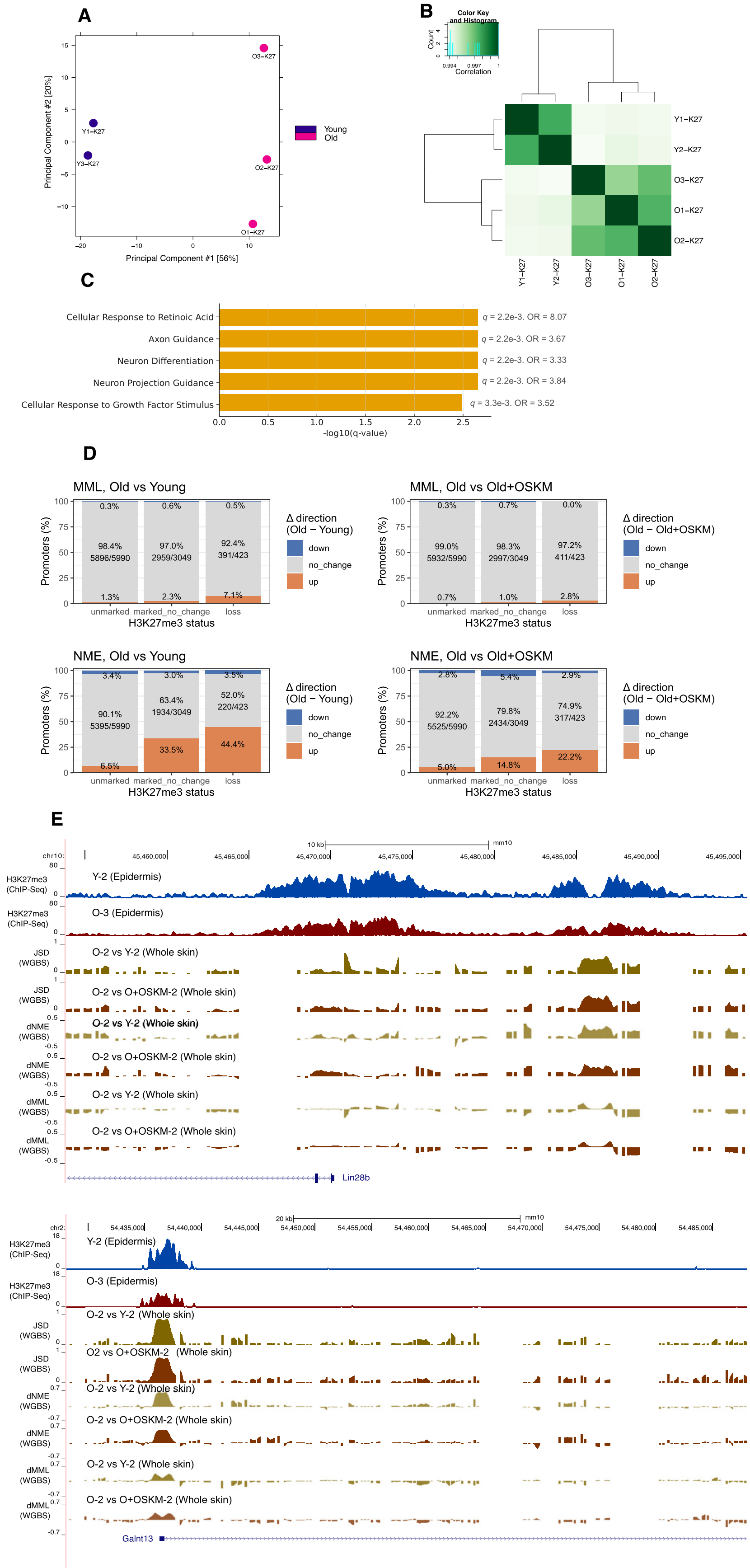
A
Principal Component #1 [56%]
Principal Component #2 [20%]
O3–K27
Y1–K27
Y3–K27
O2–K27
O1–K27
Young
Old
B
Color Key and Histogram
Count
Correlation
Y1–K27
Y2–K27
O3–K27
O1–K27
O2–K27
C
Cellular Response to Retinoic Acid
Axon Guidance
Neuron Differentiation
Neuron Projection Guidance
Cellular Response to Growth Factor Stimulus
q = 2.2e-3. OR = 8.07
q = 2.2e-3. OR = 3.67
q = 2.2e-3. OR = 3.33
q = 2.2e-3. OR = 3.84
q = 3.3e-3. OR = 3.52
-log10(q-value)
D
MML, Old vs Young
MML, Old vs Old+OSKM
NME, Old vs Young
NME, Old vs Old+OSKM
Promoters (%)
H3K27me3 status
unmarked marked_no_change loss
Δ direction (Old – Young)
Δ direction (Old – Old+OSKM)
down
no_change
up
0.3% 0.6% 0.5%
98.4% 5896/5990
97.0% 2959/3049
92.4% 391/423
1.3% 2.3% 7.1%
0.3% 0.7% 0.0%
99.0% 5932/5990
98.3% 2997/3049
97.2% 411/423
0.7% 1.0% 2.8%
3.4% 3.0% 3.5%
90.1% 5395/5990
63.4% 1934/3049
52.0% 220/423
6.5% 33.5% 44.4%
2.8% 5.4% 2.9%
92.2% 5525/5990
79.8% 2434/3049
74.9% 317/423
5.0% 14.8% 22.2%
E
H3K27me3 (ChIP-Seq)
Y-2 (Epidermis)
O-3 (Epidermis)
JSD (WGBS)
dNME (WGBS)
dMML (WGBS)
O-2 vs Y-2 (Whole skin)
O-2 vs O+OSKM-2 (Whole skin)
O2 vs O+OSKM-2 (Whole skin)
Lin28b
Galnt13
mm10

 © The Author(s)

Figure EV3. Quality assesment and integration of H3K27me3 ChIP-seq with DNA methylation.

(**A**) PCA plot of input-subtracted H3K27me3 genome coverage from young and old tail epidermis samples. (**B**) Correlation heatmap of input-subtracted H3K27me3 genome coverage from young and old tail epidermis samples. (**C**) Top five over-represented gene sets from the GO Biological Processes collection identified performing ORA via Enrichr using the genes exhibiting differential H3K27me3 at their promoter in epidermis samples during aging. (**D**) Barplots showing the proportion of promoters with CpG islands that exhibit increased, decreased, or unchanged MML (top row) and NME (bottom row), measured only within CpG islands, in old untreated (Old) vs Young and Old vs old treated (Old+OSKM) whole skin, stratified by H3K27me3 status (unmarked, marked with no change, or H3K27me3 loss) measured by ChIP on purified epidermis. Values indicate percentages and number of promoters in each category. Promoters were classified as having increased or decreased MML or NME when the difference in condition-level averages exceeded ±5% (see "Methods"). (**E**) Examples of genes exhibiting loss of H3K27me3 during aging in regions with increased DNA methylation discordance, as indicated by elevated JSD and dNME in Old untreated (Old) samples compared to Young and Old treated (Old+OSKM) samples.

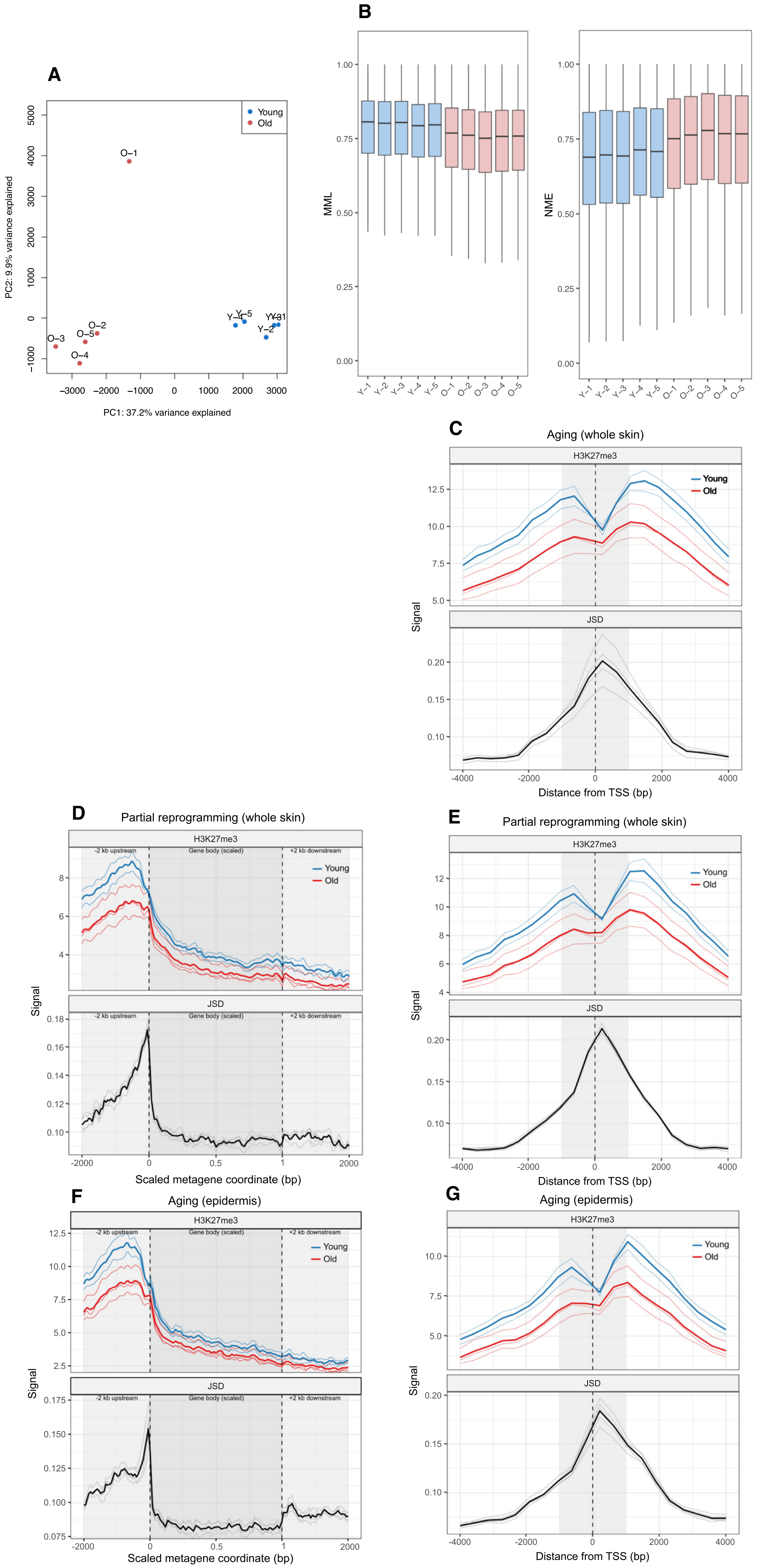
A
Young
Old
O–1
O–2
O–5
O–3
O–4
Y–4
Y–5
Y–3
Y–1
Y–2
PC2: 9.9% variance explained
PC1: 37.2% variance explained
B
MML
NME
Y–1 Y–2 Y–3 Y–4 Y–5 O–1 O–2 O–3 O–4 O–5
C
Aging (whole skin)
H3K27me3
JSD
Young
Old
Signal
Distance from TSS (bp)
D
Partial reprogramming (whole skin)
H3K27me3
JSD
-2 kb upstream
Gene body (scaled)
+2 kb downstream
Young
Old
Signal
Scaled metagene coordinate (bp)
E
Partial reprogramming (whole skin)
H3K27me3
JSD
Young
Old
Signal
Distance from TSS (bp)
F
Aging (epidermis)
H3K27me3
JSD
-2 kb upstream
Gene body (scaled)
+2 kb downstream
Young
Old
Signal
Scaled metagene coordinate (bp)
G
Aging (epidermis)
H3K27me3
JSD
Young
Old
Signal
Distance from TSS (bp)

 © The Author(s)

Figure EV4. Global DNA methylation patterns in epidermis and metaplot profiles or H3K27me3 occupancy and JSD.

(**A**) PCA plot of genome-wide MML in WGBS samples from young and old isolated tail epidermis samples. (**B**) Box plots of genome-wide MML distributions in WGBS from isolated tail epidermis of young ($n = 5$) and old ($n = 5$) mice. For each box plot, the central line represents the median, the box bounds correspond to the first (Q1) and third quartiles (Q3), and the whiskers extend to the most extreme data points within 1.5× the interquartile range (IQR) from the quartiles. (**C–G**). Metaplots of H3K27me3 occupancy and DNA methylation discordance (JSD) during aging and rejuvenation. Gene-level plots (**D**, **F**) show scaled gene bodies flanked by ±2 kb, while TSS-centered plots (**C**, **E**, **G**) show promoter-proximal regions spanning ± 4 kb around transcription start sites. Thin lines represent individual replicates for H3K27me3 ChIP, while for JSD they represent individual pairwise comparisons, where each old sample is compared against a representative control sample (aging in whole skin: Old vs Y-3; rejuvenation in whole skin: Old+OSKM vs O-2; aging in epidermis: Old vs Y-1). Bold lines represent the average signal. (**C**) TSS-centered metaplot for the set of genes with significant promoter-restricted JSD-based methylation discordance during aging in whole skin. (**D**) Gene-level metaplot for the set of genes with significant JSD-based methylation discordance over promoters and gene bodies in partial reprogramming in whole skin. (**E**) TSS-centered metaplot for the set of genes with significant promoter-restricted JSD-based methylation discordance in partial reprogramming in whole skin. (**F**) Gene-level metaplot for the set of the top 1000 JSD-ranked genes (based on discordance over promoter and gene body) during aging in purified epidermis. (**G**) TSS-centered metaplot for the set of the top 1000 JSD-ranked genes (based on discordance over promoter) during aging in purified epidermis.

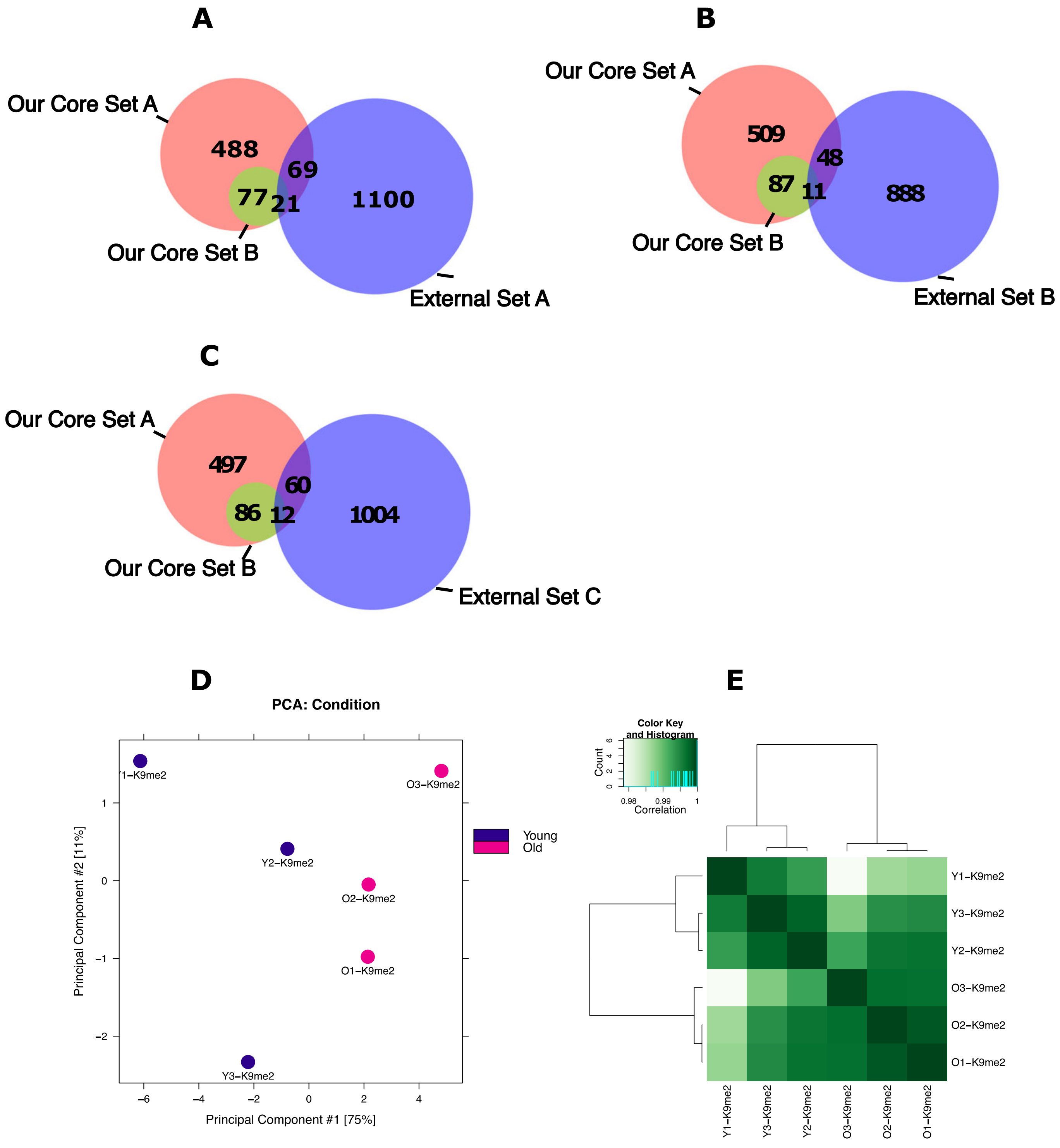

Figure EV5. Cross-study overlap of age-related epigenetically dysregulated genes and quality assessment of H3K9me2 ChIP-seq.

(**A**–**C**) Overlap between core sets of epigenetically dysregulated genes identified in this work and gene lists from other studies. 'Our Core Set A' refers to genes with DNA methylation discordance (significant JSD over promoters and gene body) in both aging and partial reprogramming-mediated rejuvenation, identified in our WGBS data. 'Our Core Set B' refers to genes included in 'Core set A' in addition to significant differences in H3K27me3 enrichment during aging, identified in our ChIP-Seq data. (**A**) Overlap with External Set A (Horvath et al, 2022a). The list is based on genes linked to CpGs significantly associated with aging in naked mole rats. (**B**) Overlap with External Set B (Moqri et al, 2024). The list is based on genes linked to 'PRC2-enriched lowly methylated regions', as defined by the authors, in human epidermis. The authors claim 90% of such regions gain DNA methylation during aging. (**C**) External Set C (Yang et al, 2023b). The list is based on genes linked to significant changes in H3K27me3 enrichment during liver aging. (**D**) PCA plot of input-subtracted H3K9me2 genome coverage from young and old tail epidermis samples. (**E**) Correlation heatmap of input-subtracted H3K9me2 genome coverage from young and old tail epidermis samples.

© The Author(s)
